# DFA-YOLO: Deformable Spatial Attention and Hierarchical Fusion for Robust Object Detection in Adverse Weather

**DOI:** 10.3390/s26072229

**Published:** 2026-04-03

**Authors:** Lu Xie, Liwen Cheng

**Affiliations:** College of Physical Science and Technology, Yangzhou University, Yangzhou 225009, China; mz120230630@stu.yzu.edu.cn

**Keywords:** object detection, adverse weather, YOLOv11, deformable convolution, attention mechanism

## Abstract

In complex real-world scenarios, object detection faces significant challenges due to severe noise interference and feature degradation. To overcome these limitations, this paper proposes DFA-YOLO, an enhanced YOLOv11 framework integrating three key innovations. First, a Deformable Spatial Attention (DSA) module is introduced into the C3k2 backbone blocks, which dynamically adjusts the receptive field to focus on informative spatial regions. This significantly enhances the model’s adaptability to geometric variations and occluded objects. Second, a Hierarchical Multi-Scale Fusion Module (HMFM) is designed to dynamically recalibrate feature responses across scales, enhancing the model’s perception of multi-scale targets. Third, an improved Wasserstein loss function combines small-object adaptive weighting with dynamic gradient modulation to address boundary ambiguity and scale sensitivity under adverse conditions. Extensive experiments on the RTTS dataset validate the superiority of our approach, achieving improvements of 3.4% and 2.8% in mAP50 and mAP50-95, respectively. Additional experiments on the Exdark dataset confirm the method’s robust generalization capability, with significant accuracy gains observed across all benchmarks.

## 1. Introduction

Road traffic safety is crucial for protecting lives and property. It is a core public concern for daily commutes and serves as a fundamental safeguard for social harmony, stability, and citizen well-being. Road object detection is a core technology in fields such as intelligent transportation and autonomous driving, where its accuracy directly impacts the reliability of downstream applications. Although object detection models demonstrate exceptional accuracy on benchmark datasets collected under ideal visual conditions, their performance often degrades significantly when deployed in adverse weather phenomena [1]. Challenges such as contrast reduction caused by dense fog, motion blur induced by heavy rain, occlusion noise resulting from snowfall, and severely constrained signal-to-noise ratios in low-light environments collectively undermine the robustness of existing detectors [2,3]. Therefore, pioneering research on visual perception under complex conditions is essential [4]. It not only provides safer perceptual safeguards for autonomous vehicles but also lays a crucial foundation for building next-generation traffic management systems resilient to challenging weather. This study focuses on enhancing the YOLOv11 model for object detection in complex environments. The improved model aims to enhance robustness against environmental changes, accurately capture object features from low-quality images, and maintain efficient inference speeds.

The work of this paper is as follows.

(1) The C3k2-DSA module is proposed by integrating deformable spatial attention into the original C3k2 bottleneck. It is designed to dynamically adjust the sampling and response regions, enabling the backbone network to adaptively extract features in complex visual scenes.
(2) A Hierarchical Multi-Scale Fusion Module (HMFM) is designed, integrating hierarchical multi-scale channel attention with enhanced spatial attention and incorporating a global context-aware mechanism. This design is aimed at effectively improving the model’s feature discrimination in complex environments characterized by low visibility and fog interference.
(3) The Small-Target Wasserstein-Adaptive WIoU Loss is proposed. Building upon the classical Wasserstein distance, this loss function incorporates small object adaptive weighting and a dynamic gradient adjustment mechanism based on Wise-IoU (WIoU). It is optimized to enhance the model’s localization robustness for small objects under poor visibility conditions, while ensuring numerical stability to maintain reliable training.

## 2. Related Work

### 2.1. Object Detection in Adverse Weather

Target detection technology under adverse weather conditions is significantly more complex than under normal conditions, primarily due to obstacles such as reduced visibility, noise interference and feature degradation [5]. To address the performance degradation of conventional detectors in harsh weather, early research typically employed image preprocessing methods. These methods processed images captured under adverse conditions prior to target detection, aiming to restore them to a clean state. Fu et al. [6]. used convolutional neural networks (CNN) to extract high-frequency rain streak information for removing existing rain streaks while restoring background details. AOD-Net [7] and FFANet [8] employed CNNs to model atmospheric scattering for direct reconstruction of clear images. However, a significant limitation of this approach is its high computational cost and the potential introduction of artifacts during restoration, which may inadvertently mislead subsequent detectors. In recent years, researchers have further explored image prior models with more physically interpretable mechanisms. Ling et al. [9] proposed utilizing Saturation Line Prior (SLP) knowledge to estimate transmittance, constructing saturation lines to address local pixel issues. Liu et al. proposed the IHDCP [10], which models transmission as a function of the inverted haze density map based on the statistical relationship between haze density and transmission. This approach enables precise capture of spatially varying haze concentration through pixel-wise gamma correction, addressing the poor generalization of traditional priors. Furthermore, approaches such as DsNet [11], TogetherNet [12] and BAD-Net [13] have established multi-task learning frameworks where image restoration and detection share features and are jointly optimized. However, this approach leads to complex network architectures and presents significant training difficulties.

Furthermore, when annotated data from clear weather is abundant but scarce for adverse conditions, domain adaptation methods offer a viable solution. These methods aim to minimize the feature distribution discrepancy across the source-target domain shift. S-DAYOLO [14] is a progressive domain-adaptive object detection framework that innovatively constructs an auxiliary domain from the source images and their style-transformed synthetic counterparts. Validation across diverse weather, lighting and sensor variation scenarios demonstrates significant improvements in cross-domain object detection performance. DSCA [15] aligns semantic information between source and target domains, supplementing and enhancing missing information in the target domain. However, domain adaptation methods involve complex alignment processes, and their performance is influenced by the degree of domain disparity.

### 2.2. Attention Mechanism

Attention mechanisms have emerged as core components within deep neural networks. By dynamically assigning weights to feature maps, they highlight key information while suppressing interference from irrelevant features, thereby significantly enhancing a model’s expressive power and robustness in complex visual tasks. Among these, the most representative attention mechanisms include channel attention [16,17], spatial attention [18] and hybrid attention [19]. As research advances, designing attention mechanisms that synergistically optimize for complex scenarios and computational efficiency has become a frontier. Addressing the challenge of varying object scales, the MSCA [20] module proposes that multi-scale information interaction and convolutional operations enable more efficient contextual encoding. It also resolves the high computational complexity of self-attention mechanisms and the issue of some models neglecting multi-scale feature aggregation. The MCA attention module [21] constructs interactions between two parallel axial attention layers, enabling more effective utilization of multi-scale features and global contextual information. FSAS [22] combines the efficiency of frequency-domain computation with the characteristics of convolutional operations, effectively reducing attention computation complexity. Dynamic sparse attention [23] achieves a balance between efficiency and accuracy by dynamically selecting important feature regions, significantly reducing computational overhead while preserving fine-grained local information.

## 3. Methodology

YOLOv11 [24] builds upon the strengths of its predecessor, YOLOv8, while introducing key architectural refinements to enhance performance. A crucial improvement is the introduction of the computationally efficient C3k2 module, which reduces latency without sacrificing feature representation. YOLOv11 incorporates a novel C2PSA module that integrates an attention mechanism to dynamically reweight feature importance. Furthermore, the detection heads are augmented with additional depthwise convolution layers to decouple and refine classification and regression features. It is particularly suitable for challenging scenarios such as adverse weather conditions, where targets are often obscured, blurred, or presented with low contrast.

**Figure 1:**
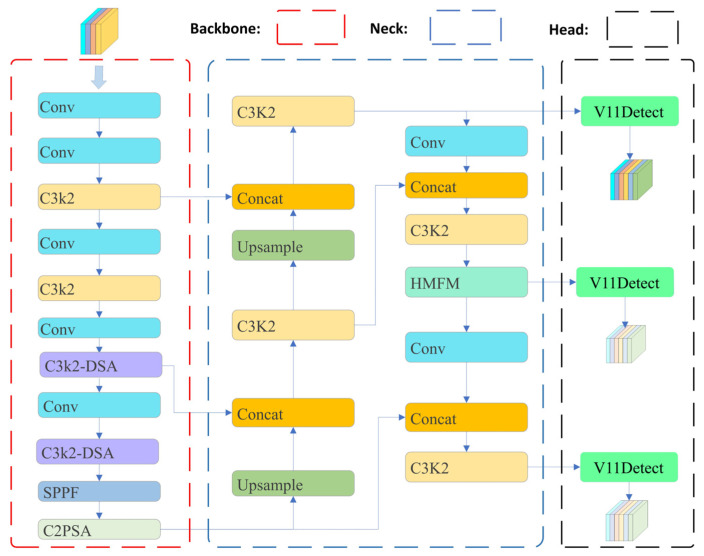
Overall structure of DFA-YOLO.

The YOLOv11 architecture encounters specific challenges in complex environments, including feature distortion, significant scale variations, and blurred object boundaries. Therefore, we propose DFA-YOLO, an enhanced detection network. Its comprehensive architecture is depicted in Figure 1. DFA-YOLO is based on a single-stage detection paradigm and primarily comprises three components: (1) a lightweight backbone network optimized for feature extraction under adverse weather conditions; (2) a neck network designed for multi-scale feature enhancement and fusion, which integrates our proposed HMFM; (3) three detection heads. Furthermore, to mitigate the issue of boundary blurring and imprecise target localization under complex scenes, we designed a Small-target Wasserstein-Adaptive WIoU loss. The following sections elaborate on the design of each key component.

### 3.1. C3k2-DSA Module

The C3k2 module in YOLOv11, while efficient, relies on fixed grid structure that struggle to address image feature degradation, severely limiting the model’s representational capabilities in complex scenes. Consequently, we integrated the DSA [25] module into the bottleneck of the C3k2 module, placing it after the second convolutional layer. This enables each bottleneck block to dynamically adjust its spatial sampling points via deformable convolutional kernels, thereby adaptively focusing on critical regions.

**Figure 2:**
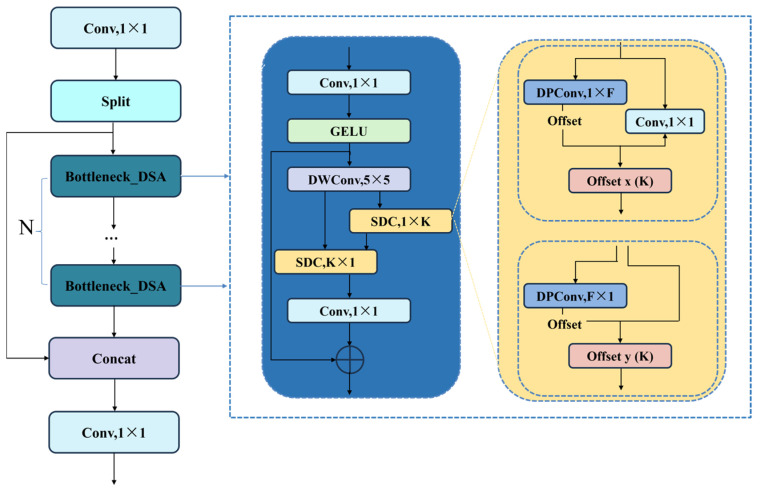
Structure of the C3k2-DSA. ‘Offset x’ and ‘Offset y’ denote the convolutional layers learning spatial offsets for horizontal and vertical deformations, respectively.

The core innovation of the DSA module lies in efficiently integrating strip-shaped deformable convolutions with a spatial gating mechanism. As shown in Figure 2. Structure of the C3k2-DSA., the input feature map is first projected through a 1 × 1 convolutional layer, then refined via a GELU activation function. The features are then fed into the spatial gating unit, whose core innovation lies in decomposing traditional deformable convolutions into two independent deformable convolutions along the x-axis and y-axis. These deformable convolutions enable the model to adaptively sample feature maps, focusing on important regions. The operations for strip-shaped deformable convolutions along the x-axis and y-axis are as follows:

(1) $$y^{1}\left(i_{0},j_{0}\right)=\sum_{g=1}^{G}\sum_{j=0}^{K_{w}-1}w\cdot \mathrm{Lin}\left(x,i_{0},j_{0}+j+∆p_{j}^{g}\right)$$

(2) $$y^{2}\left(i_{0},j_{0}\right)=\sum_{g=1}^{G}\sum_{j=0}^{K_{h}-1}\mathrm{Lin}\left(y^{1},i_{0}+i+∆p_{i}^{g},j_{0}\right)$$

where *Lin* denotes linear interpolation, $∆p_{j}^{g}$ and $∆p_{i}^{g}$ represent learnable offsets along the x-axis and y-axis, respectively. $K_{w}$ and $K_{h}$ denote the number of sampling points in the x and y directions for the convolution kernel. G represents the number of deformable groups.

Then the two deformable paths are fused through element-wise multiplication to form a coherent spatial attention map. The final output is obtained via a residual connection that adds the original input to the enhanced features. This preserves important low-level information and stabilizes training. The C3k2-DSA enables the model to effectively overcome the receptive field limitations of fixed-grid convolutions and learn more discriminative spatial attention maps.

### 3.2. Hierarchical Multi-Scale Fusion Module

**Figure 3:**
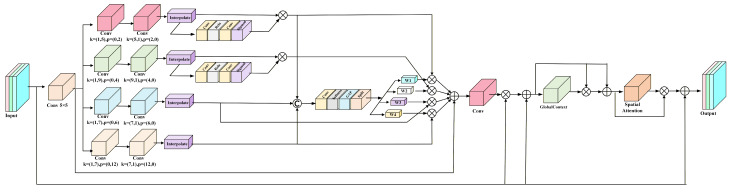
Structure of the HMFM.

In severe weather, noise interference severely weakens feature extraction, which fails to enhance both the distinctiveness of global features for large objects and the local details for small objects simultaneously. While existing attention mechanisms like CBAM [19] and ASFF [26] have been widely employed to enhance models’ focus on critical regions, their designs often rely on single-scale modeling. This makes it difficult to accommodate the wide range of scale variations from small pedestrians to large vehicles. Traditional methods typically rely on fixed-rate dilated convolutions or simple pyramid pooling, which may not align with the irregular scale distribution of objects in complex weather contexts. Therefore, we propose a hierarchical multi-scale fusion module. By capturing features of targets at different scales through four parallel branches, it explicitly constructs a receptive field pyramid that spans from local to global. This design enables the network to adaptively assign the most suitable feature extraction scale for objects of varying sizes, rather than forcing all features through a bottleneck of uniform scale. Figure 3 illustrates the structure of HMFM.

Given an input feature map $X\in R^{B\times C\times H\times W}$, preliminary feature extraction is first performed using a 5 × 5 DSConv to obtain the foundational features. For small objects, heterogeneous convolution kernels of size (1, 5) and (5, 1) are employed to capture elongated edge structures along the horizontal and vertical axes, thereby avoiding the redundant computation associated with full-kernel convolutions. For medium-sized objects, kernels with wider receptive fields (1, 9) and (9, 1) are utilized to further expand the lateral semantic coverage. To capture the overall contours of distant or large objects, dilated convolutions are introduced.

(3) $$\tilde{X}=\delta \left(BN\left({DSConv}_{5\times 5}\left(X\right)\right)\right)$$

where δ  denotes the ReLU activation, *BN* is batch normalization. Considering that small-scale features are more prone to losing detail in severe weather, we introduced a lightweight gating mechanism after the small-scale and mesoscale feature paths.

(4) $${\hat{F}}_{small}=F_{small}⨀\sigma \left({Conv}_{1\times 1}\left(\delta \left({Conv}_{1\times 1}\left(F_{small}\right)\right)\right)\right)$$

(5) $${\hat{F}}_{medium}=F_{medium}⨀\sigma \left({Conv}_{1\times 1}\left(\delta \left({Conv}_{1\times 1}\left(F_{small}\right)\right)\right)\right)$$

where σ  denotes the Sigmoid function, ⨀  denotes element-wise multiplication.

Feature maps from four scales are concatenated along the channel dimension and processed through a weight generator. This generator consists of a 1 × 1 convolution, a Softmax function and a global average pooling layer, which collectively produce a set of spatial weights relevant to the input content. This design allows for the autonomous adjustment of multi-scale feature fusion weights according to the target scale, which can be formulated as:

(6) $$W_{s}=\mathrm{GAP}\left(\mathrm{Softmax}\left({\mathrm{Conv}}_{1\times 1}\left(\left[{\hat{F}}_{small};{\hat{F}}_{medium};F_{large};F_{xlarge}\right]\right)\right)\right)$$

where $\left[\cdot \right]$ denotes channel concatenation, $\mathrm{GAP}\left(\cdot \right)$ represents global average pooling.

To perform weighted fusion, the spatial attention weight map $W_{s}$ is proportionally divided into four distinct weighted maps: $W_{s}$, $W_{m}$, $W_{l}$, and $W_{xl}$, each corresponding to a specific feature scale. Channel consistency is then restored through a 1 × 1 convolution, producing the final channel attention feature $A_{c}$ as follows:

(7) $$A_{c}={\mathrm{Conv}}_{1\times 1}\left[\tilde{X}+\sum_{s\in \left\{S,M,L,XL\right\}}W_{s}⨀F_{s}\right]$$

Channel attention maps are combined with the original input through residual connections. Subsequently, we introduced a global context module. This module strengthens the global information representation of large targets through global average pooling and a channel compression expansion mechanism, as follows:

(8) $$X^{\prime }{=A}_{c}⨀X+X$$

(9) $$G=\sigma \left(MLP\left(\mathrm{GAP}\left(X^{\prime }\right)\right)\right)$$

(10) $$X\prime \prime =X^{\prime }⨀G+X^{\prime }$$

Next, spatial attention $A_{s}$ is optimized by aggregating average and max-pooled features, with the calculation formula as follows:

(11) $$A_{s}=\delta \left({\mathrm{Conv}}_{7\times 7}\left(\mathrm{Concat}\left(\mathrm{AvgPool}\left(X\prime \prime \right),\mathrm{MaxPool}\left(X\prime \prime \right)\right)\right)\right)$$

The final output of the HMFM is shown in Equation (12):

(12) $$Y=X\prime \prime ⨀A_{s}+X$$

The HMFM employs multi-scale recalibration across different spatial locations and feature channels. This hierarchical design provides greater flexibility compared to the original single-scale processing. The global context enhancement module strengthens the utilization of global information through a lightweight channel compression and expansion mechanism without significantly increasing computational load. Furthermore, the introduction of multi-statistical spatial attention enables the model to perceive not only the mean and extreme values of features but also the dispersion of their distributions. This provides richer discriminative information especially when handling targets with blurred edges or complex textures.

### 3.3. Small-Target Wasserstein-Adaptive WIoU Loss

The original YOLOv11 network employed a combination of binary cross-entropy (BCE) [27] for classification, distributed focal loss (DFL) [28] for bounding box regression and complete intersection over union (CIoU) [29]. Under complex weather conditions, traditional IoU-based regression losses are prone to gradient vanishing or oscillation due to minor positional deviations. They also exhibit insufficient scale sensitivity, particularly yielding poor regression performance for small targets and low-quality samples. Wasserstein distance [30] measures the divergence between two probability distributions, offering a smoother reflection of the spatial relationships between bounding boxes. This property makes it especially suitable for optimizing the localization of small and blurry objects. Nevertheless, the standard Wasserstein loss lacks sensitivity to extremely small targets and suffers from numerical instability in high-noise environments. Furthermore, in complex weather scenarios, gradient allocation between difficult and easy samples requires finer-grained control.

Therefore, we propose a Small-target Wasserstein-Adaptive WIoU loss (SWAWIoU). This loss function is enhanced based on the Wasserstein distance metric by introducing an adaptive weighting mechanism for small objects and a dynamic gradient modulation strategy for WIoU [31], thereby constructing a robust loss function suitable for complex weather scenarios. SWAWIoU not only effectively enhances sensitivity for small targets but also guarantees numerical stability during training, making it widely applicable for multi-scale detection under complex conditions.

To establish a differentiable and geometrically meaningful similarity measure between bounding boxes beyond Intersection over Union (IoU), we propose to represent each box as a second-order Gaussian distribution. Specifically, the predicted box $B_{p}=\left(c_{x}^{p},c_{y}^{p},w_{p},h_{p}\right)$ and the ground truth box $B_{g}=\left(c_{x}^{g},c_{y}^{g},w_{g},h_{g}\right)$,where (cx, cy) represents the center coordinates, w and h denote the width and height.

When the covariance matrix is diagonal, the second-order Wasserstein distance formula simplifies into an efficient and numerically stable expression that simultaneously accounts for both center distance and size variation. Calculation formula:

(13) $$W_{2}^{2}={\left(c_{x}^{p}-c_{x}^{g}\right)}^{2}+{\left(c_{y}^{p}-c_{y}^{g}\right)}^{2}+{\left(\frac{w_{p}}{2k}-\frac{w_{g}}{2k}\right)}^{2}+{\left(\frac{h_{p}}{2k}-\frac{h_{g}}{2k}\right)}^{2}$$

where k is a scaling factor controlling the sensitivity of the Wasserstein distance to bounding box dimensions, set to 2. To alleviate the difficulty of detecting small targets under adverse weather conditions and enhance the model’s sensitivity, we introduced a scale-sensitive enhancement factor γ, calculated as follows:

(14) $$\gamma =exp\left(-\frac{w^{g}{\cdot h}^{g}}{1000}\right)$$

Considering the significant area differences, an adaptive penalty term has been introduced. $P_{area}$ is defined as follows:

(15) $$P_{area}=\frac{\left|w^{p}\cdot h^{p}-w^{g}{\cdot h}^{g}\right|}{w^{g}{\cdot h}^{g}}\cdot \gamma$$

The enhanced Wasserstein distance incorporates area penalties and supports controllable scaling. Calculation formula:

(16) $${\tilde{W}}_{2}^{2}=W_{2}^{2}+\lambda \cdot min\left(P_{area},\kappa \right)$$

where λ is the equilibrium hyperparameter controlling the strength of area penalty, set to 0.5. κ is the upper bound threshold preventing gradient explosion caused by outlier samples, set to 10.

Traditional Wasserstein distance loss employs a fixed scaling constant. We propose an adaptive scaling mechanism calculated as follows:

(17) $$C^{\prime }=C_{0}\cdot \left(1+\frac{\gamma }{2}\right)$$

where $C_{0}$ is the baseline scaling constant, defaulting to 12.8. This design effectively attenuates the normalized intensity for small objects, thereby enhancing the relative weight of their distance signals within the loss function.

To convert this augmented distance into a similarity metric, we employ an exponential transform with adaptive scaling. The adaptive scaling constant is dynamically adjusted based on object size according to the following formulation:

(18) $$S=exp\left(-\frac{\sqrt{{\tilde{W}}_{2}^{2}}}{C^{\prime }}\right)$$

**Figure 4:**
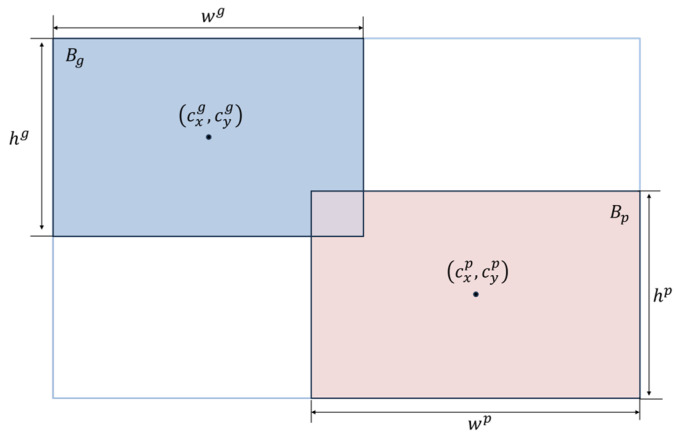
Geometric illustration of intersection over union.

We employ the dynamic non-monotonic focusing mechanism from WIoU. This mechanism constructs a gradient attention weight inversely proportional to the outlierness of the Intersection over Union (IoU), effectively reducing the influence of low-quality anchor boxes to the total loss and mitigating the harmful gradients they produce. To intuitively illustrate the computational principles and geometric significance of IoU, Figure 4. Geometric illustration of intersection over union. depicts a visual representation of their spatial overlap. The IoU is calculated as follows:

(19) $$IoU=\frac{Area\left(B_{p}⋂B_{g}\right)}{Area\left(B_{p}⋃B_{g}\right)}$$

The gradient modulation factor η is calculated as follows:

(20) $$\eta =\frac{{\left(1-IoU\right)}^{\beta }}{E\left[{\left(1-IoU\right)}^{\beta }\right]+\varepsilon }$$

where β = 1.5 controls the focus on hard examples. $E\left[\cdot \right]$ denotes the in-batch expected moving average estimate.

The final loss function formula is shown in Equation (21).

(21) $$L=\eta \cdot \left(1-S\right)$$

The SWAWIoU loss is optimized for complex detection scenarios. It incorporates a target-adaptive enhancement mechanism that automatically identifies targets and amplifies their corresponding gradient responses. The area matching penalty term introduces a proportional constraint to synergistically optimize positional accuracy and scale alignment, thereby guiding the model to predict bounding boxes with more appropriate dimensions. A weighted fusion strategy effectively combines the IoU with the area penalty, balancing the objectives of localization accuracy and size estimation through carefully calibrated coefficients. Furthermore, a dual dynamic modulation mechanism retains the advantages of the original WIoU dynamic scaling while introducing dynamic adjustment based on area information. This multi-level adaptive strategy allows the loss function to respond to the varying demands of different training phases and object characteristics.

## 4. Experiments and Results

### 4.1. Experimental Datasets

We utilized two challenging adverse weather datasets. The first is the annotated Real-world Task-driven Testing Set (RTTS) [32], which comprises 4322 images that primarily depict real-world foggy driving scenarios. The visual samples are annotated with bounding boxes for five common road object categories: car, bicycle, motorcycle, pedestrian, and bus. In addition, we assessed the generalization capability of DFA-YOLO through evaluations on the ExDark [33] dataset. The ExDark dataset is the largest curated collection of low-light images, consisting of 7363 samples across 12 distinct lighting conditions. These datasets were all divided into training, validation and test sets in an 8:1:1 ratio for experimental purposes.

### 4.2. Experimental Environment and Evaluation Metrics

The proposed algorithm was implemented and trained on a remote computational server using the PyTorch 2.0 framework [34]. Table 1 presents the specifications of the experimental setup. The model was trained for 200 epochs with a batch size of 8. The optimizer was SGD, configured with an initial learning rate of 0.01, momentum of 0.937, and weight decay of 0.0005. All input images were resized to a fixed resolution of 640 × 640 pixels. To enhance the generalization capability of the model, the mosaic data augmentation technique was integrated into the training pipeline.

**Figure 5:**
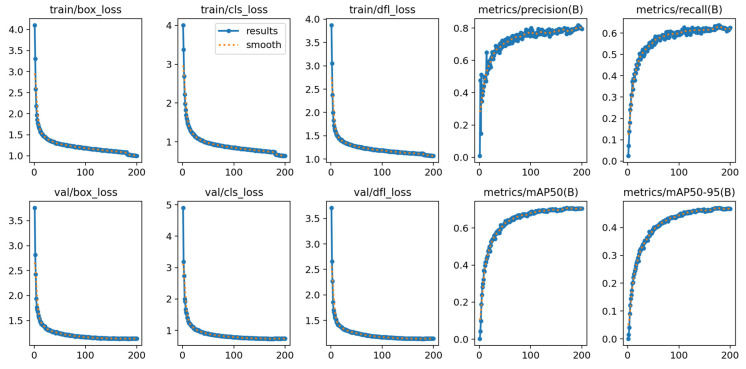
Training performance metrics.

Figure 5 illustrates the training process of DFA-YOLO on the RTTS dataset. It demonstrates a significant downward trend in multiple loss functions. This indicates the model’s sustained and stable convergence on the training set, confirming effective optimization in localization, classification and distribution prediction capabilities. On the validation set, all loss functions similarly maintained a stable decline, verifying the model’s strong generalization capability without noticeable overfitting. Concurrently, the model’s detection precision and recall rates steadily improved, reflecting significantly enhanced recognition and localization capabilities for positive samples. The steady increase in the mean average precision (mAP) metric indicates that the model maintains consistent detection stability across different IoU thresholds. The synchronized improvement in loss curves and accuracy curves indicates that the enhanced YOLOv11 model exhibits favorable convergence characteristics and learning stability during training. Although minor fluctuations occurred in certain metrics like precision and recall during the latter stages of training, the overall trend aligns with expectations, validating the effectiveness of the proposed improvement strategy.

The detection performance is quantified using precision (P), recall (R) and mAP, while model parameter count (Params) and computational complexity (GFLOPs) serve as measures of efficiency and computational cost. Additionally, inference latency defined as the end-to-end time required for the model to process a single image.

Precision refers to the proportion of true positives among the results predicted as positive by the model. Recall refers to the proportion of all true positives that are successfully predicted as positive by the model. mAP is uesd to assess a model’s aggregate performance across all categories. It is calculated by computing the Average Precision (AP) for each category and then averaging these AP values. The specific calculation formula is as follows:

(22) $$Precision=\frac{TP}{TP+FP}$$

(23) $$Recall=\frac{TP}{TP+FN}$$

(24) $$AP=\frac{1}{m}\sum_{i}^{m}P_{i}=\int P\left(R\right)dR$$

(25) $$\;mAP=\frac{1}{N}\sum_{j}^{N}AP_{j}$$

### 4.3. Ablation Experiment

To systematically evaluate the effectiveness of DFA-YOLO in enhancing target recognition accuracy in complex environments and to rigorously assess the independent and synergistic contributions of each innovative component, we conducted ablation experiments on the RTTS and ExDark datasets.

The original YOLOv11n architecture as the baseline, we employed a controlled variable approach to sequentially and combinatorially introduce three core improvement modules. The experimental setup is as follows: (1) single-module validation to access the performance gains from each independent module; (2) dual-module combination to explore the synergistic effects between the C3k2-DSA module and HMFM; (3) full model integration to validate the combined performance of all components. All experiments were conducted under identical training protocols, hyperparameter configurations, datasets and evaluation protocols to ensure the comparability of results. Detailed quantitative results are summarized in Table 2, where “✔” indicates the module was used.

First, by integrating the C3k2-DSA module independently into the baseline model, mAP@50 improved from 72.3% to 73.1%, while the params and GFLOPs remained comparable to YOLOv11. The result confirms that the C3k2-DSA module effectively guides the model to focus on key spatial features obscured by haze. In contrast, using the HMFM alone can boost mAP@50 to 73.8%, despite a moderate computational overhead. This trade-off remains acceptable in practical applications, underscoring the module’s efficacy in improving cross scale feature aggregation. The independent application of the SWAWIoU loss function also improved accuracy, demonstrating that optimizing the bounding box regression process enhances localization precision. Subsequently, we investigated the synergistic effects of the combined modules. Experimental results clearly validate the effectiveness of each innovative module and their synergistic interactions, robustly demonstrating the superiority of DFA-YOLO in improving object detection robustness under complex environmental conditions. Although the proposed DFA-YOLO exhibits a slight increase in parameter count and inference latency, this trade-off is entirely justified for adverse weather perception tasks that prioritize robustness.

We conducted a comprehensive evaluation of DFA-YOLO on ExDark dataset by progressively integrating individual design components. The results are detailed in Table 3. From the baseline to enabling all methods, mAP@50 improved from 56% to 59.6%, while mAP@50–95 increased from 34.4% to 36.4%. The results demonstrate that even in complex scenarios or under special lighting conditions, the C3k2-DSA module can still extract more robust features. The HMFM significantly compensates for insufficient low-level texture information caused by inadequate illumination through hierarchical processing and multi-strategy fusion, ultimately boosting all performance metrics. For potentially complex object locations in the Exdark dataset, SWAWIoU enables more precise adjustment of the prediction boxes, thereby improving localization accuracy.

**Figure 6:**
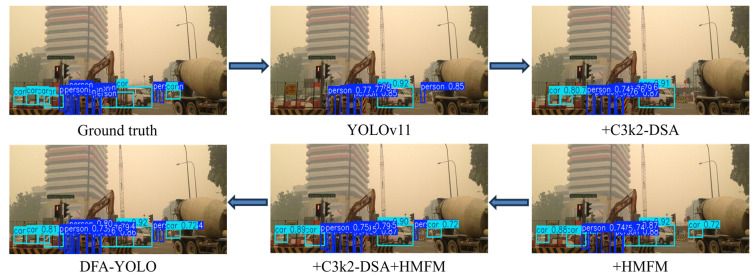
Visual comparison of different ablation settings.

To further illustrate the effectiveness of different components, representative visual comparisons under different ablation settings are presented in Figure 6. The baseline detector tends to miss small or low-contrast objects under challenging conditions. After introducing the proposed modules, the detector can better capture object features and produce more accurate bounding boxes. In particular, the complete model achieves more reliable detections and reduces missed detections compared with other ablation variants.

Precision and recall exhibit an intrinsically inverse relationship, which is comprehensively characterized by the Precision–Recall curve. Its area under the curve serves as a robust indicator of the detector’s performance across varying confidence thresholds, with curves closer to the top-right corner reflecting superior classification efficacy for a given category.

**Figure 7:**
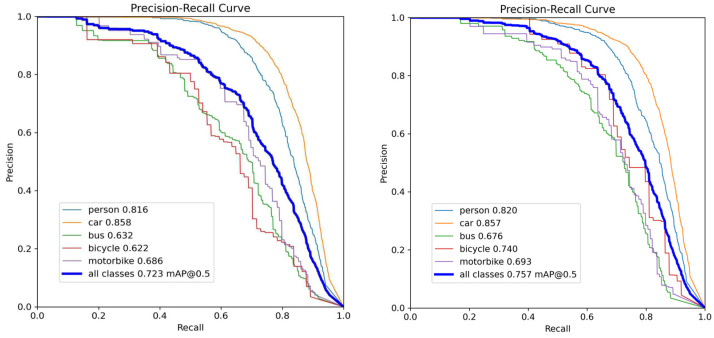
P-R curve of all class for YOLOv11n and DFA-YOLO on RTTS.

**Figure 8:**
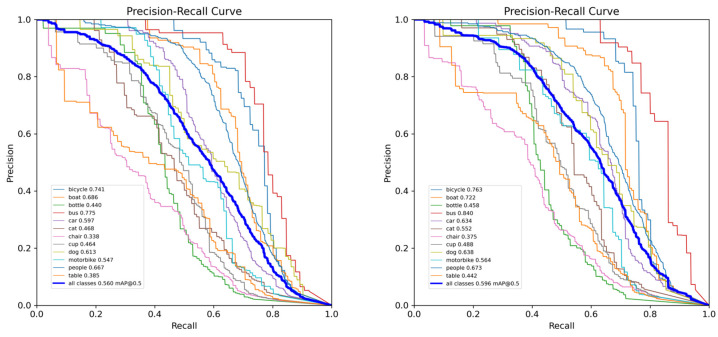
P-R curve of all class for YOLOv11n and DFA-YOLO on Exdark.

Figure 7 and Figure 8 present the P-R curves of YOLOv11 and the proposed DFA-YOLO on the RTTS and Exdark datasets, respectively. It can be observed that across both datasets, the DFA-YOLO curves for all categories significantly outperform YOLOv11. This demonstrates a marked improvement in the overall detection capability of our method under diverse confidence threshold settings, underscoring its enhanced discriminative power and robustness in complex environmental conditions.

To rigorously evaluate the superiority of the proposed Small-target Wasserstein-Adaptive WIoU loss, we conducted comparisons against various loss functions. The experiments utilized our established architecture integrating C3k2-DSA and HMFM. This analysis aims to quantify the performance improvements conferred by our loss function under challenging weather conditions. The SWAWIoU demonstrates superior performance to other loss on both key evaluation metrics, mAP50 and mAP50-95. Specifically, its mAP50 score improves by 1.8% compared to the baseline Wasserstein loss function, as detailed in Table 4.

### 4.4. Compared with Other SOTA Models

We evaluated multiple object detection networks under consistent experimental conditions and parameter settings, as summarized in Table 5 and Table 6. The compared methods encompass a diverse range of architectures, including representative one-stage detectors (e.g., SSD [35], RetinaNet [36], and YOLO series [37,38]), two-stage detectors (e.g., Faster R-CNN), and transformer-based detectors (e.g., RT-DETR [39] and Deformable DETR [40]). Recent advanced YOLO variants, such as Hyper-YOLO [41] and IA-YOLO [42]. Furthermore, to assess the impact of image enhancement on detection performance under foggy and low-light conditions, we evaluated two-stage pipelines that integrate enhancement networks, specifically AOD-Net [7] and Zero-DCE [43], with Faster R-CNN. Among all compared methods, DFA-YOLO demonstrates strong detection capabilities in complex environments.

### 4.5. Visual Analysis

**Figure 9:**
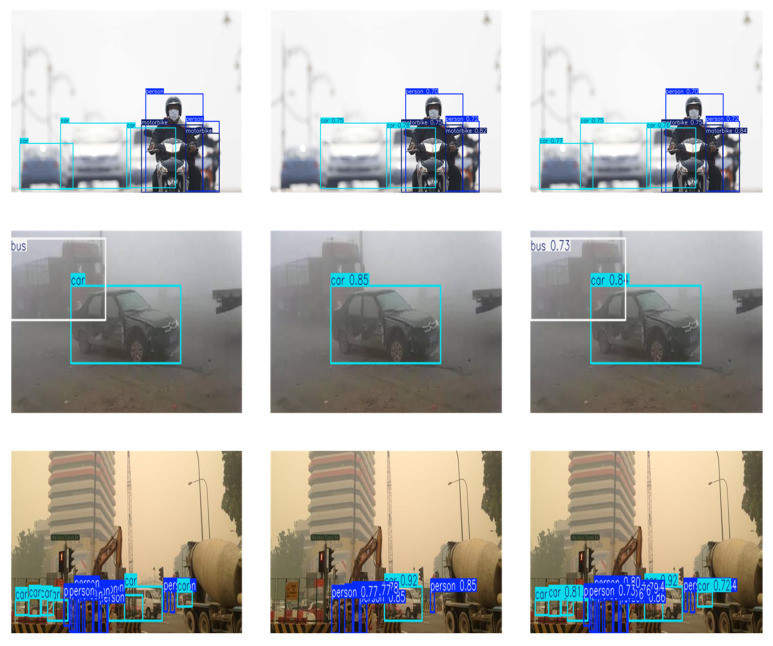
Detection results of YOLOv11 and DFA-YOLO.

Finally, to visually compare the detection performance, we randomly sampled images from the datasets. Figure 9 presents a comparative visualization, showing from left to right: the manually annotated ground truths, the results of YOLOv11 and the results from the proposed DFA-YOLO. Clearly demonstrate that DFA-YOLO achieves significantly superior detection performance than the baseline model YOLOv11. In adverse weather scenarios, YOLOv11 exhibits noticeable missed detections. For instance, in the first and second rows of images, YOLOv11 missed one vehicle in each; the third row shows that YOLOv11 failed to detect multiple distant objects in the image. In contrast, DFA-YOLO demonstrates a significantly lower error rate.

**Figure 10:**
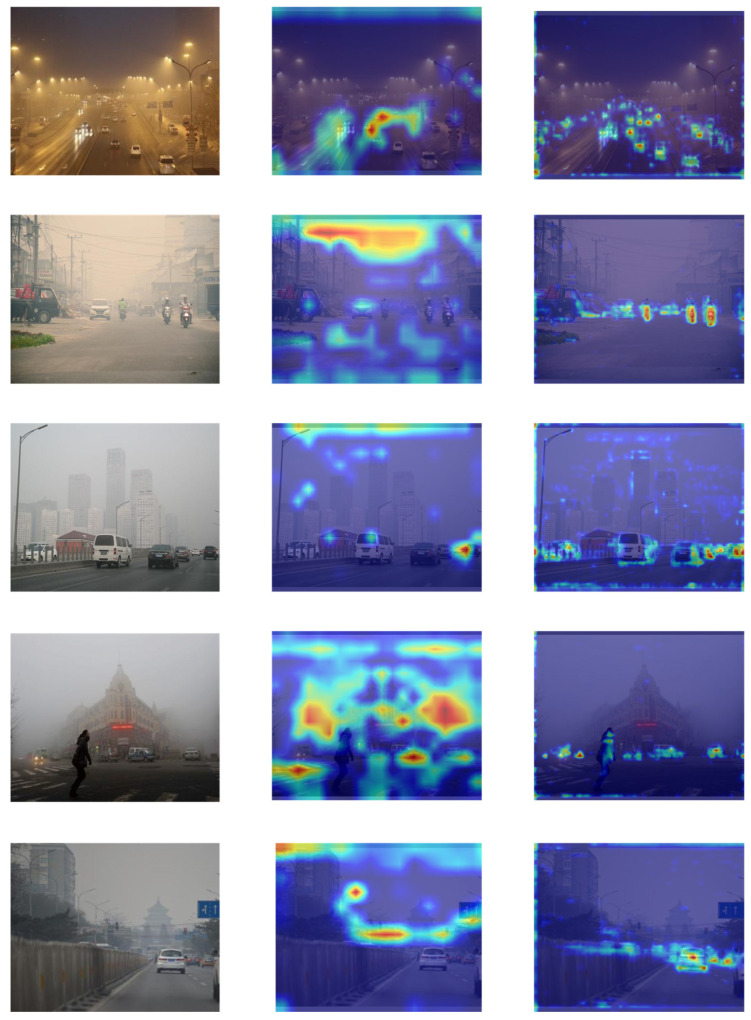
Grad-CAM visualization.

Additionally, Grad-CAM [44] heatmaps are used for intuitive visualization, as shown in Figure 10. The left column displays the original image, the middle column shows the Grad-CAM heatmap for YOLOv11, and the right column for DFA-YOLO. These visualizations clearly demonstrate that the YOLOv11 model is more susceptible to environmental interference, resulting in insufficient feature learning. In contrast, DFA-YOLO by integrating the Deformable Spatial Attention module and a hierarchical multi-scale feature fusion approach, focuses more effectively on the critical feature regions of target objects. This improved focus on key regions reduces the impact of environmental noise and leads to more precise object localization.

**Figure 11:**
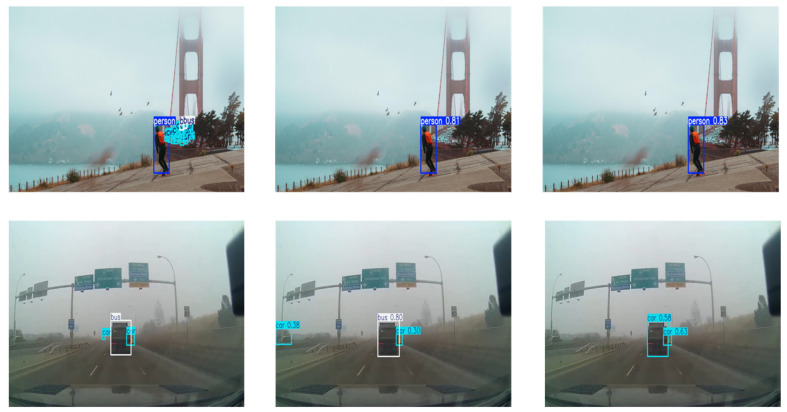
Failure comparison.

Despite the progress made by DFA-YOLO, certain limitations persist, particularly in the detection of extremely small objects. As illustrated in Figure 11, pedestrians or vehicles are frequently missed or mislocalized. This issue primarily stems from two factors: first, the severe degradation of spatial details caused by adverse weather, which disproportionately affects small objects; and second, the inherent difficulty of feature extraction at extremely low resolutions—even advanced attention mechanisms struggle to extract discernible information from such data. These challenges warrant continued attention in future research.

## 5. Conclusions

This paper proposes an improved YOLOv11 architecture to address the challenges of object detection in complex scenes. Through synergistic design across three dimensions—feature extraction optimization, feature enhancement mechanisms, and loss function innovation, we introduce a novel backbone enhancement by integrating a deformable spatial attention into the C3k2 module. This overcomes limitations of traditional convolutions by enabling flexible spatial sampling and adaptive receptive fields, thereby boosting backbone performance. The HMFM is embedded within the neck network of DFA-YOLO. This integration equips the network with the ability to adaptively perceive and fuse multi-scale contextual information, enabling precise detection of objects across varying scales in complex weather conditions. To tackle the difficulties in detecting small objects under complex conditions such as high miss rates and positioning inaccuracies caused by environmental interference, this paper proposes an enhanced Wasserstein loss function. The function integrates adaptive weighting for small objects, numerical stability enhancement and dynamic gradient adjustment mechanisms. It significantly improves model robustness in adverse weather while maintaining detection accuracy. Comprehensive experiments conducted on two challenging datasets demonstrate that the proposed DFA-YOLO achieves substantial improvements in detection accuracy under adverse conditions. Exhibiting strong generalization capabilities, DFA-YOLO effectively balances the critical trade-offs among accuracy, efficiency and robustness, offering a reliable perception solution for complex real-world autonomous driving environments.

## Figures and Tables

**Table 1:** Experimental environment configuration.

Parameters	Configuration
CPU	15 vCPU Intel(R) Xeon(R) Platinum 8362 CPU @ 2.80 GHz
GPU	RTX 3090 (24 GB)
System environment	Ubuntu 20.04
Framework	PyTorch 2.0
Programming voice	Python 3.8.10
Cuda	11.8

**Table 2:** Comparison of RTTS dataset ablation experiments.

Base	C3k2-DSA	HMFM	SWAWIoU	mAP@50	mAP@50-95	P	R	GFLOPs	Params (M)	Time (ms)
✔				72.3	48	77.3	62.8	6.3	2.58	7.9
✔	✔			73.1	48.3	76.3	63.6	6.3	2.54	9.2
✔		✔		73.8	48.7	79.3	63.6	6.8	2.69	8.0
✔			✔	73.5	48.6	76.3	63.2	6.3	2.58	7.5
✔	✔	✔		74.3	49.3	79.6	65.9	6.6	2.68	10.2
✔	✔	✔	✔	75.7	50.8	80.3	67.3	6.6	2.68	10.0

**Table 3:** Comparison of Exdark dataset ablation experiments.

Base	C3k2-DSA	HMFM	SWAWIoU	mAP@50	mAP@50-95	P	R	GFLOPs	Params (M)	Time (ms)
✔				56	34.4	65.4	50.8	6.3	2.58	5.2
✔	✔			56.5	34.5	66.1	51.4	6.3	2.54	6.4
✔		✔		58.3	35.9	68.9	52.6	6.8	2.69	6.1
✔			✔	58.1	35.5	72.4	51.6	6.3	2.58	4.9
✔	✔	✔		58.6	35.7	69.2	52.1	6.6	2.68	6.8
✔	✔	✔	✔	59.6	36.4	71.9	53.6	6.6	2.68	6.6

**Table 4:** Comparison of loss function.

Evaluation Index	CIoU	EIoU	SIoU	WIoU	ShapeIoU	MPDIoU	NWD	Ours
mAP50	74.3	74	73.8	74.9	74.2	75.2	73.9	75.7
mAP50-95	49.3	49.2	48.9	50	48.1	49.9	49.7	50.8

**Table 5:** Comparing the results of different algorithm models on the RTTS dataset.

Model	P	R	mAP50	mAP50-95	GFLOPs
SSD	77.9	48.4	63.1	37.4	30.63
Faster R-CNN	77.4	57.3	69.3	43.7	37.54
AOD+Faster R-CNN	77.5	57.2	67.6	43.1	37.54
IA-YOLO	51.1	36.3	38.2	21.7	/
RetinaNet	67.2	62.1	64.8	38.4	170.1
RT-DETR	71.0	59.6	65.7	43.0	103.5
Deformable DETR	72.8	64.7	71.8	47.2	160
YOLOv5	74.9	64.3	72.6	48.1	7.1
YOLOv8	78.1	66.4	72.3	48.8	8.1
YOLOv10	80.5	60.8	72.4	47.3	6.5
YOLOv11	77.3	62.8	72.3	48.0	6.3
hyper-YOLO	78.9	67.9	73.7	49.6	9.7
Ours	80.3	67.3	75.7	50.8	6.6

**Table 6:** Comparing the results of different algorithm models on the Exdark dataset.

Model	P	R	mAP50	mAP50-95	GFLOPs
SSD	56.9	38.4	45.1	23.4	30.63
Faster R-CNN	65.4	57.3	54.3	30.7	37.54
Zero-DCE+Faster R-CNN	65.1	56.9	54.1	30.1	37.54
IA-YOLO	56.6	49.2	40.7	23.6	/
RetinaNet	58.2	50.1	48.4	25.3	170.1
RT-DETR	67.8	52.6	58.2	36.4	103.5
Deformable DETR	66.4	50.1	56.3	34.6	160
YOLOv5	65.6	51.5	52.4	28	7.1
YOLOv8	69.8	51.1	58.4	35.4	8.1
YOLOv10	67.6	50.2	55.6	33.8	6.5
YOLOv11	65.4	50.8	56.0	34.4	6.3
hyper-YOLO	69.8	53.8	59.9	36.6	9.7
Ours	71.9	53.6	59.6	36.4	6.6

## Data Availability

The datasets used in this study are publicly available. The RTTS dataset can be accessed at https://www.kaggle.com/datasets/tuncnguyn/rtts-dataset (accessed on 20 June 2025), and the Exdark dataset is available at https://github.com/cs-chan/Exclusively-Dark-Image-Dataset (accessed on 20 June 2025). All data used in the analysis are contained within the article.
